# The impact of mean body mass index on reported mortality from COVID-19 across 181 countries

**DOI:** 10.3389/fpubh.2023.1106313

**Published:** 2023-03-13

**Authors:** Ruggero Gabbrielli, Nicola Maria Pugno

**Affiliations:** ^1^Laboratory for Bioinspired, Bionic, Nano, Meta Materials and Mechanics, Department of Civil, Environmental and Mechanical Engineering, University of Trento, Trento, Italy; ^2^School of Engineering and Materials Science, Queen Mary University of London, London, United Kingdom

**Keywords:** body mass index, COVID-19, mortality, public health, overeating

## Abstract

Accountability for global health issues such as a pandemic and its devastating consequences are usually ascribed to a virus, but a comprehensive view should also take into account the state of the host. Data suggests that excessive nutrition is to blame for a yet unknown but not negligible portion of deaths attributed to severe acute respiratory syndrome coronavirus 2. We analyzed the correlation between mean body mass index (BMI) and 2-year coronavirus disease 2019 (COVID-19) mortality rates reported by 181 countries worldwide. Almost two thirds of the countries included had a mean BMI greater or equal to 25, with death rates ranging from 3 to 6,280 per million. Death rates in countries with a mean BMI below 25 ranged from 3 to 1,533. When the analysis was restricted to countries where the extent of testing was deemed more representative of actual mortality, only 20.1% had a mean BMI <25 but the mortality difference persisted. A second analysis looking at pre-vaccination mortality obtained from a different source led to similar conclusions. Due to the nature of the variables, reverse causation can be excluded while common causation can not. A mean BMI <25 for a country seems to spare its citizens from the highest COVID-19 mortality rates. The impact of excess weight on global COVID-19 mortality is suspected to have been much higher than what currently perceived, here estimated at no less than a fourfold increase in mortality. Countries with normal mean BMI constitute precious test beds for the quantification of the effects of overeating on COVID-19 mortality.

## Introduction

Koch's third postulate states that in order to establish a causal relationship between a microbe and a disease, the microorganism should cause disease when introduced into a *healthy organism*. No reference is made to the severity of the disease caused, nor to what constitutes a healthy organism, nor to the possibility for the organism to be in a state of partial well-being.

Hill improved upon Koch's original criteria in many aspects, but the difficulties encountered in the quantification of the initial state of health of the host and how this could affect both infectivity and mortality remain. An organism can at any time and for a variety of different reasons weaken a subset of its own defense systems, with no apparent ill effects. It is only when a new threat that specifically exploits that very weakness that the host begins to tumble.

For this reason we asked ourselves whether the COVID-19 pandemic—had the virus not found a species afflicted by overeating—would have been as deadly as it had.

Obesity, once considered by many clinicians a self-inflicted condition of little medical significance, has increased dramatically during the last four decades (1). Today we know that it carries higher risks for the development of type 2 diabetes, coronary heart disease, a number of cancers, respiratory complications and osteoarthritis. Even more modest degrees of overweight are associated with mortality (2). Health care resources are inundated by obesity and its consequences (3), with high social and economic costs, including attempts to prevent or to treat it (4).

Although failure to mention obesity as one of the preexisting diseases associated with death still occurs (5), individuals with obesity, overall and central, are more at risk for being COVID-19 positive [46% more (6)] (7–9), hospitalization (10), ICU admission (6, 9), reinfection (11) and mortality [48% more (6)] (7). Obesity and impaired metabolic health are important risk factors for severe COVID-19 (12–16). The risk of hospital admission or death due to COVID-19 starts at a body mass index (BMI) as low as 23 kg/m^2^ (13). Central obesity and hypertension are associated with lower antibody titres in response to COVID-19 mRNA vaccine (17). Hypertension was found to be more prevalent in the first surge of the disease in Iran, where patients were also younger (18). SARS-CoV-2 infection induces neutralizing antibodies only in few obese COVID-19 patients (19). Given that BMI has a causal role in the development of severe COVID-19, the promotion of weight loss in people with obesity or overweight would help to combat the COVID-19 pandemic (12). In addition to increased risks for the subject, people with high BMI were also found to transmit the infection more easily. The number of exhaled respiratory droplets were in fact considerably higher for these individuals, and further increased with degree of COVID-19 infection (20). A positive association with death was found in previously hospitalized individuals for BMI >37 (21) and among infected underweight, obesity class II and III patients (22).

Among the very old, overweight and obesity were found not to be associated with in-hospital mortality (23). For patients aged ≥60 year, mild/moderate obesity was associated with a 13% reduced mortality risk and a 10% increased length of stay in the ICU (24). Although underweight patients had a higher risk of mortality from COVID-19 (25), overweight and obese patients outnumber them in most countries, making an analysis of mean values not futile.

The combination of increased infection rates, increased transmissibility and decreased antibody response to vaccine, all lead to a larger basic reproduction number. Quantification of the mutual amplification of such effects remains a challenge. Since the majority of the population in many high-income countries is overweight, and since both overweight adolescents and adults experience more respiratory symptoms (26), the role played by excess weight on the intensity of the initial viral diffusion and on its temporal and spatial evolution across the globe should not be ignored.

At this time, doubt pushed us to explore the patterns of mortality around the globe, and their relation to BMI, trying to go beyond both the limitations of BMI itself (definitions of overweight range from BMI ≥23 to BMI ≥25) and our struggle with estimations of exact death tolls (27). In the era of globalization, a survey of this kind might not come as handy as one might expect, but if interpreted correctly it is still revealing.

## Materials and methods

The first problem we encountered was to assess data comparability. Since under-testing and under-reporting of deaths is believed to be common in developing countries, we initially considered analyzing the correlation between COVID-19 tests per capita and reported COVID-19 deaths. The analyses included over 180 countries for which both BMI and mortality data were present. We then conducted very basic observations on the patterns of correlation between COVID-19 mortality and mean BMI, plus three additional variables that for different reasons we felt could potentially have an influence: cigarette consumption [chronic obstructive pulmonary disease worsens outcomes from COVID-19 (28)], annual average precipitation and average temperature (29, 30). Additional indices were looked at, such as the gross domestic product at purchasing power parity, but it was felt that its impact on mortality, although significant, required a socioeconomic type of analysis, which was beyond the scope of this work.

Data for each country was initially collected from a number of different sources and collated into a single file. This included mean BMI from the World Health Organization's Global Status Report on noncommunicable diseases (WHO) (31), population, COVID-19 deaths and tests from the global statistics provider Worldometers (32) and the project Our World in Data (33) (OWID), life expectancy at birth in 2019 from WHO, annual cigarette consumption per person from the Tobacco Atlas (34), annual average precipitation from the World Bank (35) and average temperature from the Climatic Research Unit (36). Data extracted from OWID, in addition to deaths and tests, included the day COVID-19 statistics recording began. This was functional to derive annual quantities, considering that different countries started official recording at different times.

**Figure 1:**
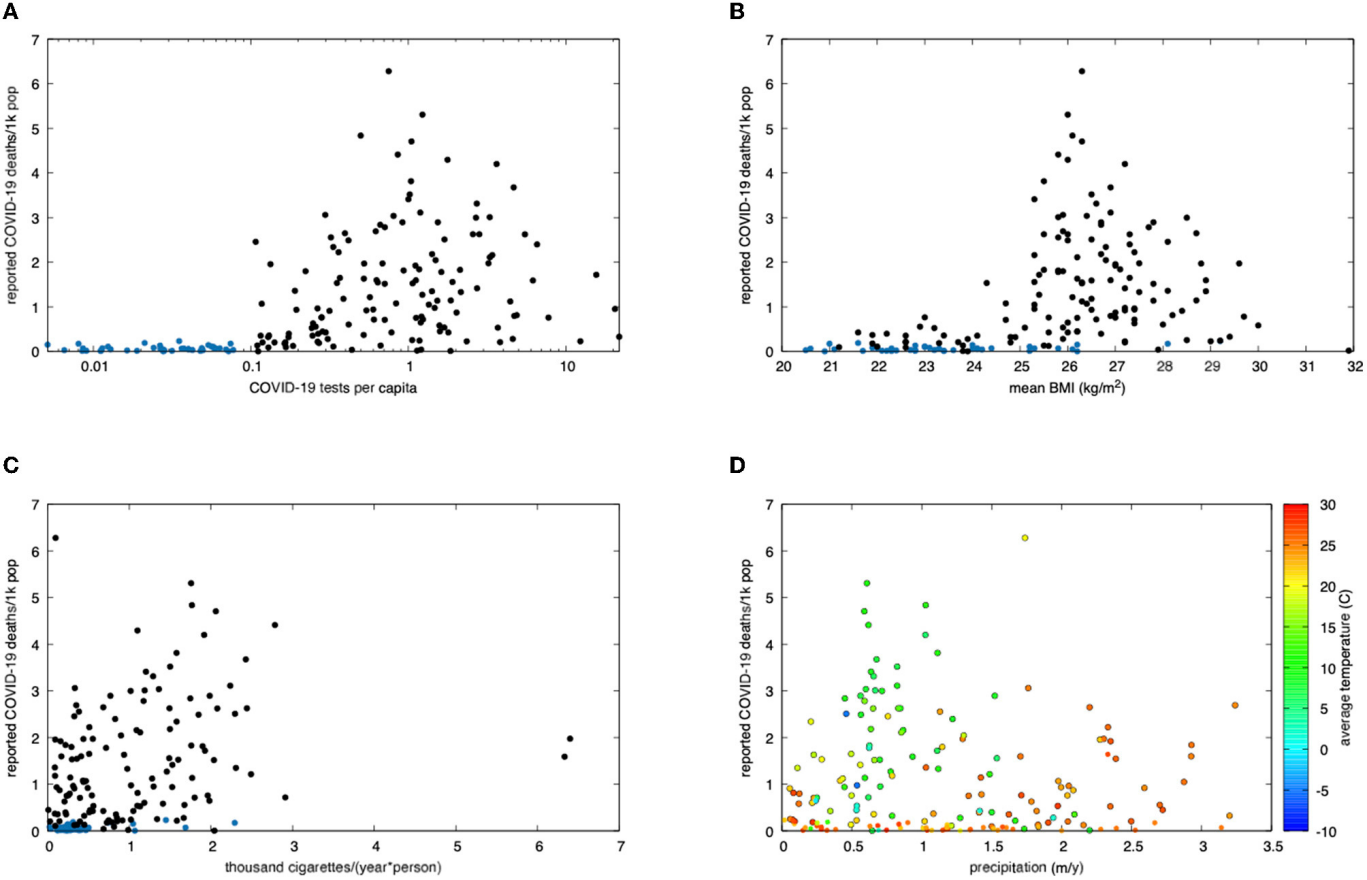
Correlation plots analysing COVID-19 mortality as a function of BMI, cigarette consumption, annual precipitations and average temperature in 181 countries worldwide COVID-19 data covers about 2 years, from initial counting until 25 March 2022. Purportedly under-reporting countries are shown in blue in the first three panels and edgeless in the last one. **(A)** Scattered plot of reported COVID-19 deaths vs. tests per capita (worldometers.info, retrieved on 25 March 2022). Under-reporting of deaths seems more likely below about 50 tests per thousand people per year. **(B)** Scattered plot of reported deaths per thousand due to COVID-19 (worldometers.info) vs. mean BMI (31). **(C)** Scattered plot of reported deaths per thousand due to COVID-19 vs. cigarette smoking (tobaccoatlas.org, 2016). **(D)** Average annual precipitation (35) and temperature (1961–1990 data, Climatic Research Unit, 2011). Warmer and humid areas experienced reduced mortality. Data source: Mitchell, T.D., Carter, T.R., Jones, P.D., Hulme, M., New, M., 2003: A Comprehensive Set of High-Resolution Grids of Monthly Climate for Europe and the Globe: the Observed Record (1901–2000) and 16 Scenarios (2001–2100). J. Climate: submitted.

A first correlation between BMI and COVID-19 deaths was evidenced by data retrieved from WHO and the statistics provider Worldometer. Countries with limited testing also reported few deaths. To limit the risk of introducing less reliable data, we restricted our analysis to countries who tested beyond a specific value. This value was chosen based on the distribution of the data itself. No sharp increase in mortality was present for an increment in testing rate up to about 0.1 tests per capita (50 tests per thousand people per year). The analysis was therefore split into two groups: one containing countries who tested up to 0.1 per capita (41) and the other containing countries who tested more (134). Six countries did not have data on the number of administered tests and were excluded (Figure 1A).

In order to eliminate the effect of vaccination, and also as a mean of comparison and validation against the first database, a second COVID-19 database was used. This was freely available from OWID. For each country, the day data were first recorded was extracted. This start date was used to compute the interval of time across which mortality was reported. Total COVID deaths, tests and population of each country were also extracted. The analysis was restricted to year 2020. Using a combination of basic command line tools and a spreadsheet, a data file was generated. Three more columns were added: one for death rates, one for test rates and one for BMI. The World Health Organization's Global Status Report on noncommunicable diseases 2014 contains both 2010 and 2014 BMI data, which show increases of up to 2% over a period of 4 years, hovering around 1% for most countries. Given that COVID-19 struck 6 years later, and that adult BMI has and is seeing a linear increase worldwide (37), our analysis underestimates the effect of BMI on mortality. Regarding causality, reverse causation is for temporality reasons not possible. Common causative factors—if any is present—would call for a proper quantification of their individual impact.

Out of 214 countries, 31 had no BMI data, thus allowing analysis of 183 countries in total. All data files are available as Supplemental material.

## Results

Plotting each country's COVID-19 death rate vs. its mean BMI gave us some elementary clues (Figure 1B). After over 2 years into the pandemic, countries with a BMI < 25 reached a maximum of 1,535 deaths (Namibia), as opposed to those exceeding 25, where death rates soared, up to 6,280 (Peru, see Supplemental material). Correlation with cigarette smoking was much weaker, as shown in Figure 1C. The effect of climate is shown in Figure 1D, where both precipitations and higher average temperatures confirmed to result into a somewhat lower mortality.

**Figure 2:**
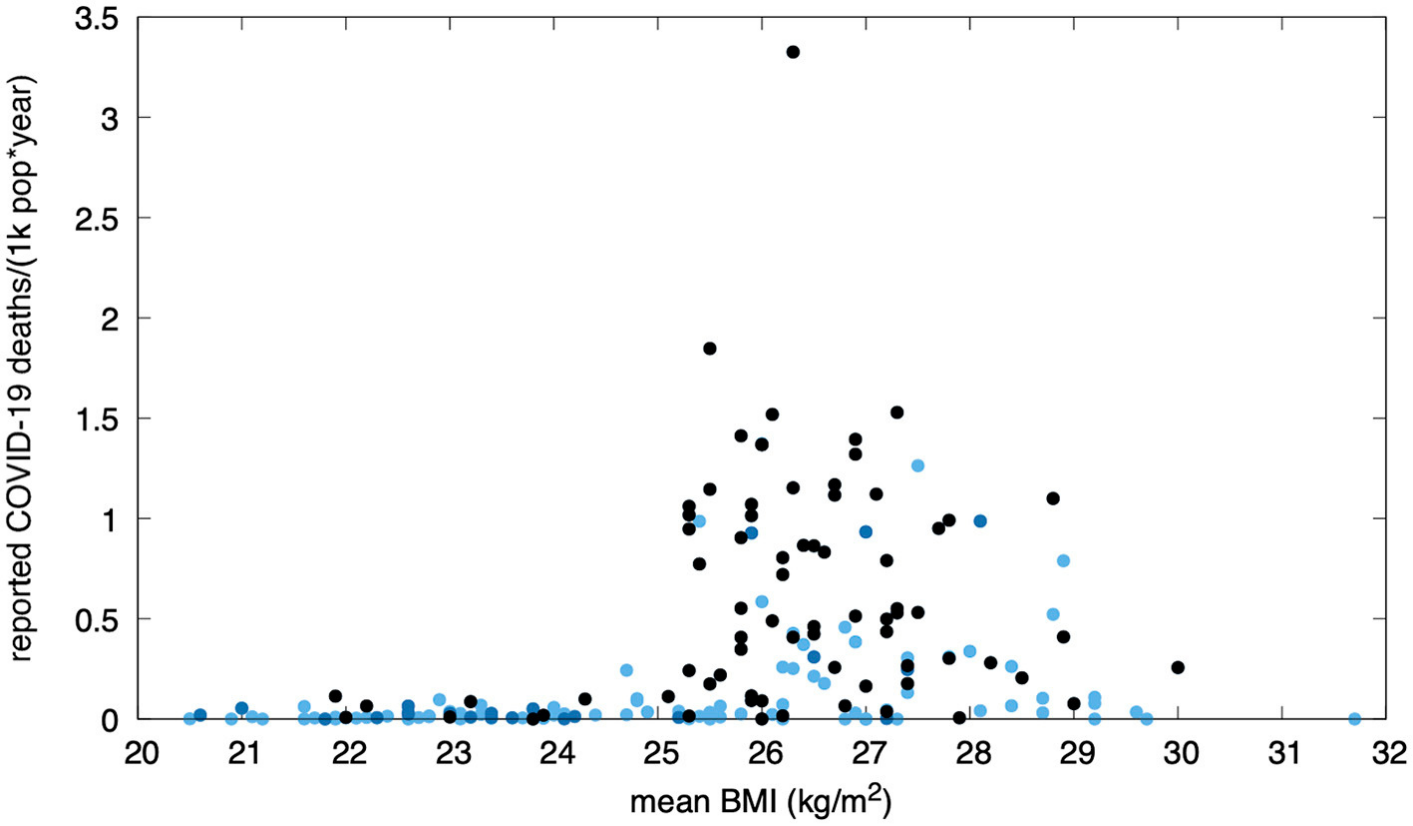
Pre-vaccination annual reported COVID-19 mortality in 183 countries (Our World in Data, year 2020) vs. mean BMI. Black dots correspond to countries who performed more than 50 tests per thousand people per year. Dark blue dots indicate countries who did not reach such a rate. Countries for which testing data was not available within this dataset are shown in light blue. The lower mortality rates among countries with a normal mean BMI can not be explained by undertesting alone (a few extensively-testing countries can be seen in this range).

COVID-19 reported death rates for year 2020, virtually free from the effects of vaccination, are shown in Figure 2. Mean mortality in normal mean BMI countries was 4.8 (1755/369, Worldometer) and 13.7 (685/50, OWID) times smaller than that found in high mean BMI countries.

**Figure 3:**
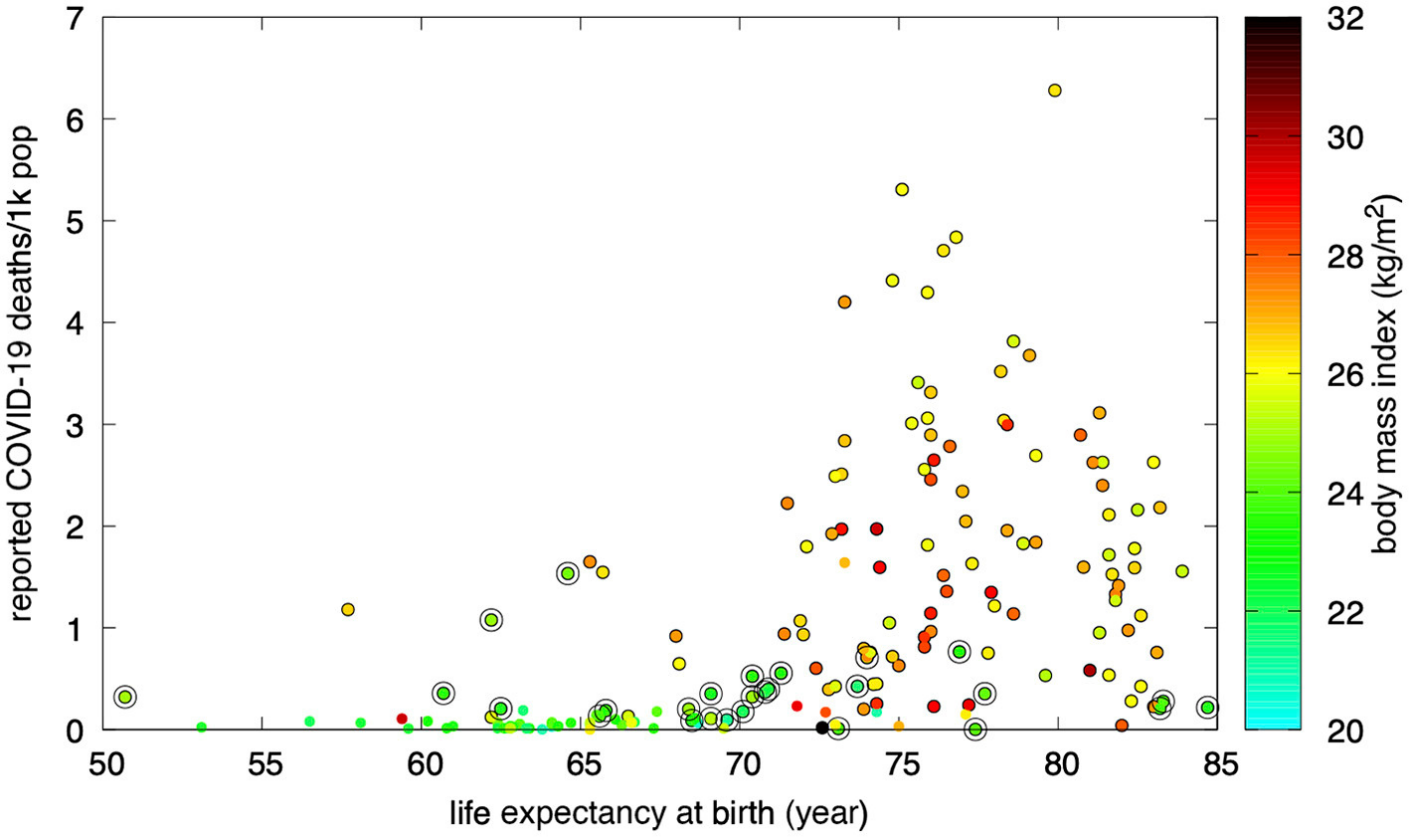
Countries with a normal mean BMI (circled) reported a lower COVID-19 mortality. Purportedly under-reporting countries are shown with edgeless dots.

A view at the recognized major risk factor for death due to COVID-19, age, can be observed in Figure 3. Countries with longer life expectancies at birth were, as expected, hit the hardest. However, countries with a mean BMI < 25 consistently showed a reduced mortality, which remained particularly low even among the few countries with the highest life expectancies.

The number of countries with a mean BMI < 25 amounts to 27 (20.1% of the total of the 134 considered). The overall distribution of mortality in this group scales down to less than one fourth of that seen for BMI ≥25. The odds ratio between the odds of a lower mortality rate in presence of a BMI < 25 and the odds of a lower mortality rate in the absence of a BMI < 25 is identically equal to 1.0 (24:24) when a reference value of 720 deaths per 1,000,000 population is taken. Defining “high reported mortality” as the mortality exceeding such a value, only 3 countries had high mortality in the presence of a BMI < 25, against 83 countries with high mortality in the absence of a BMI < 25. The resulting odds ratio was therefore 0.036 (3:83).

According to worldwide reported deaths due to COVID-19, high reported mortality in the presence of a mean BMI < 25 was rare. Countries with a mean BMI < 25 where the extent of testing was deemed representative showed less than one fourth the mortality observed in countries with a BMI ≥25.

## Discussion

Two major limitations of this study are the uncertainties on undertesting and underreporting of deaths. Lower income countries typically tested less extensively and were less likely to report deaths from COVID-19 with the needed accuracy. Actual mortality was higher than reported mortality in every part of the world, but the ratio between the two was correlated with income. Developing countries—the majority of which also have a lower mean BMI—had a much higher actual mortality compared to reported counts. This effect was, expectedly, less prominent in higher income countries. Although such a difference could have in principle represented a bias toward an overestimation of the effect of BMI on mortality, the absence of extensively-testing (high-income), normal-BMI countries reporting high mortalities remains.

Another limitation came from differential testing policy, which was not considered. However, although local and temporally delimited differences in country-specific decisions on testing symptomatic individuals only vs. everyone existed, governmental directions changed over time in most countries, making annual statistics less sensitive to this type of inequality.

As a simple anecdote, we would like to draw the reader's attention to the fact that a naked man walking in the rain in the winter is not unlikely to catch a cold. Although colds are often caused by a virus, we do not usually point the finger at the infective agent. We agree instead that the cause of his cold was his behavior, since he—aware of the risk—exposed himself to the elements. Indeed the use of face masks, social distancing and mass vaccinations, all offer protection against infection. However, limiting vulnerability to the new threats that day after day nature manages to assemble behind the scenes would not be inadvisable.

One potentially successful strategy, and a suggestion for an open medical ethics debate, would be that of entitling those who treat their body with more respect (such as normal-weight, non-smokers individuals) to small deductions on the contribution that in many countries patients owe for the cost of medical tests and treatments. An action of this kind has the potential to mitigate the obesity and smoking prevalence. It would simultaneously provide positive feedback to those who already look after their body, and would gradually awake the interest of those who struggle making an effort to change their attitude towards smoking and/or food. A rewarding scheme recognizing body respect would invert the current global trend of increasing obesity rates, simultaneously resulting in a significant reduction in public health expenditure.

## Conclusion

A higher mortality from COVID-19—up to fourfold and over—was observed in many countries where the mean BMI exceeded the value of 25, while such increase was not seen in the few extensively-testing, normal-mean-BMI countries.

Caution should nevertheless be exercised in the interpretation of the reported correlations when attempting a causal analysis. Although both a coincidence and reverse causation can be excluded due to the correlation being statistically significant and temporally unidirectional, mean values do not contain information about distribution. A country with a bimodal distribution of BMI (for example were many people are underweight and obese) could have a similar mean BMI of a country were most people have a normal BMI. Finally, a high BMI does not represent neither a necessary nor a sufficient condition for death due to COVID-19. Common causation can not be ruled out.

As with any morphometric index, BMI comes with issues. International variations are well known, such as a reduced upper normal value for Asians (38). A more promising index seems to be the dimensionless waist-to-height ratio, in short WHtR, for which at the time of this writing a global report is still missing.

## Data availability statement

The original contributions presented in the study are included in the article/Supplementary material, further inquiries can be directed to the corresponding author.

## Author contributions

RG wrote the manuscript, retrieved the data, and prepared the figures. NMP revised the manuscript, analyzed data comparability criteria, encouraged discussions, and elaborated on correlates. All authors contributed to the article and approved the submitted version.
