# Supplementary material for: The impact of mean body mass index on reported mortality from COVID-19 across 181 countries
Source: Front Public Health. 2023 Mar 13;11:1106313. doi: 10.3389/fpubh.2023.1106313 (PMC10042264; doi:10.3389/fpubh.2023.1106313)
Supplement: Supplementary file 1 [file Data_Sheet_1.PDF]

Country	BMI	COVIDdeathsp1M	cigspypp	WHOlifeexpb	COVIDtestsp1M	COVIDcasesp1M	precip	temp
**Afghanistan**	21.6	189	311.6	63.2	21160	3925	327	12.6
**Albania**	26.1	1215	2491.6	78.0	569929	71662	1485	11.4
**Algeria**	26.2	152	1046.7	77.1	5119	4773	89	22.5
**Andorra**	27.5	1975	6398.3	-	3225381	271957	-	7.6
**Angola**	24.1	55	340.2	63.1	40024	1923	1010	21.55
**Antigua and Barbuda**	28.1	1359	89.2	76.5	190517	42354	1030	26.0
**Argentina**	27.7	2783	1176.1	76.6	708534	117785	591	14.8
**Armenia**	26.7	2895	1985.7	76.0	917563	115785	562	7.15
**Australia**	27.2	225	917.0	83.0	2346981	9994	534	21.65
**Austria**	25.4	1717	1927.0	81.6	15602507	138074	1110	6.35
**Azerbaijan**	27.4	939	1525.6	71.4	597967	59465	447	11.95
**Bahamas**	28.8	1969	438.6	73.2	524011	57960	1292	24.85
**Bahrain**	28.2	813	1101.5	75.8	4876138	155808	83	27.15
**Bangladesh**	21.0	174	744.1	74.3	74327	9465	2666	25.0
**Barbados**	28.7	1142	277.5	76.0	1858859	94543	1422	26.0
**Belarus**	26.6	717	2911.3	74.8	1237917	72664	618	6.15
**Belgium**	25.5	2626	2440.9	81.4	2569422	172944	847	9.55
**Belize**	28.9	1595	168.6	74.4	1115913	76662	1705	25.3
**Benin**	23.4	13	204.0	63.4	47871	1979	1039	27.55
**Bhutan**	23.8	11	-	73.1	1851402	3390	2200	7.4
**Bolivia**	25.9	1798	291.6	72.1	222502	47587	1146	21.55
**Bosnia and Herzegovina**	26.1	4838	1767.2	76.8	498187	87892	1028	9.85
**Botswana**	24.7	1076	433.5	62.2	835311	84546	416	21.5
**Brazil**	25.9	3060	333.5	75.9	296709	103437	1761	24.95
**Brunei**	26.2	450	9.7	74.3	1585481	34755	2722	26.85
**Bulgaria**	26.0	5307	1757.3	75.1	1229280	105767	608	10.6
**Burkina Faso**	22.1	17	408.1	62.7	11168	767	748	28.29
**Burundi**	20.9	3	239.4	63.8	27773	1824	1274	19.8
**Cambodia**	21.9	178	726.2	70.1	163817	7060	1904	26.8
**Cameroon**	24.4	70	171.0	62.4	63498	3926	1604	24.6
**Canada**	27.2	975	1021.3	82.2	1469709	49610	537	-5.35
**Cape Verde**	24.7	708	397.1	74.0	707957	98767	228	23.30
**Central African Republic**	22.4	23	213.2	53.1	16382	2415	1343	23.3
**Chad**	22.3	11	279.3	59.6	11145	333	322	26.55
**Chile**	27.8	2894	769.3	80.7	1539120	92669	1522	8.45
**China**	23.9	3	2043.0	77.4	111163	70	645	6.95
**Colombia**	25.9	2693	351.4	79.3	620628	98862	3240	24.5
**Comoros**	24.1	177	585.4	67.4	-	5216	900	25.55
**Congo**	23.3	67	195.2	64.7	59474	3355	1646	-
**Costa Rica**	26.9	1597	411.4	80.8	627792	110132	2926	24.8
**Croatia**	25.5	3815	1578.9	78.6	1039918	166793	1113	10.9
**Cuba**	26.2	752	233.5	77.8	1118993	85207	1335	25.2
**Cyprus**	27.0	758	1961.4	83.1	7760297	119644	498	18.45
**Czech Republic**	26.9	3676	2427.9	79.1	4663361	224958	677	7.55
**Denmark**	25.3	951	1298.0	81.3	20508953	109996	703	7.5
**Djibouti**	23.3	187	785.5	65.8	270682	13416	220	28.0
**Dominica**	27.0	871	147.4	-	2023593	88999	2083	22.35

**Dominican Republic**	26.7	396	218.9	72.8	267506	37419	1410	24.55
**DR Congo**	22.2	14	128.0	62.4	9022	719	1543	24.0
**East Timor**	21.2	95	-	69.6	166690	14634	1500	25.3
**Ecuador**	27.0	1956	93.5	78.4	133147	29776	2274	21.85
**Egypt**	29.2	231	1449.4	71.8	35048	3578	18	22.1
**El Salvador**	27.4	629	206.7	75.0	248275	18599	1784	24.45
**Equatorial Guinea**	25.6	123	328.2	62.2	185304	9261	2156	24.55
**Eritrea**	20.5	28	132.3	64.1	6538	2150	384	25.5
**Estonia**	25.5	1829	1759.9	78.9	2128255	175749	626	5.1
**Ethiopia**	20.6	62	115.3	68.7	36347	3165	848	22.2
**Fiji**	27.2	919	491.1	68.0	541537	58115	2592	24.4
**Finland**	25.9	537	1098.8	81.6	1691974	39848	536	1.7
**France**	25.3	2159	1089.9	82.5	3412167	131952	867	10.7
**Gabon**	25.5	131	451.2	66.5	666252	16429	1831	25.05
**Gambia**	24.0	144	249.1	65.5	53734	3995	836	27.5
**Georgia**	27.2	4200	1917.7	73.3	3634650	229702	1026	5.8
**Germany**	26.3	1525	1599.5	81.7	1064309	81282	700	8.4
**Ghana**	24.2	45	40.5	66.3	69642	4131	1187	27.2
**Greece**	27.3	2624	2078.6	81.1	5495137	100283	652	15.4
**Grenada**	27.0	1922	158.2	72.9	1109400	52256	2350	26.65
**Guatemala**	26.5	934	111.8	72.0	194466	33900	1996	23.45
**Guinea**	22.7	32	316.0	61.0	42544	2257	1651	25.7
**Guinea-Bissau**	23.1	83	25.3	60.2	58079	3171	1577	26.75
**Guyana**	26.3	1546	359.1	65.7	639475	49097	2387	26.0
**Haiti**	24.1	71	143.0	64.1	11394	2234	1440	24.9
**Honduras**	26.4	1068	469.8	71.9	117195	37378	1976	23.5
**Hungary**	26.3	4706	2060.3	76.4	1046742	127841	589	9.75
**Iceland**	25.9	281	848.1	82.3	4602998	61583	1940	1.75
**India**	21.9	368	89.3	70.8	520185	24825	1083	23.65
**Indonesia**	22.9	555	1675.5	71.3	257683	15340	2702	25.85
**Iran**	26.2	1630	936.5	77.3	521584	72173	228	17.25
**Iraq**	28.0	602	1184.4	72.4	417698	50336	216	21.4
**Ireland**	27.5	1330	976.5	81.8	2153667	131786	1118	9.3
**Israel**	26.3	1121	1280.7	82.6	4436346	145558	435	19.2
**Italy**	26.0	2627	1493.3	83.0	2820779	89595	832	13.45
**Ivory Coast**	23.6	29	352.7	62.9	49511	2274	1348	26.35
**Jamaica**	27.4	963	312.5	76.0	264941	30901	2051	24.95
**Japan**	22.6	218	1583.2	84.7	265266	13741	1668	11.15
**Jordan**	28.9	1349	2306.1	77.9	1412700	100490	111	18.3
**Kazakhstan**	27.4	713	1800.9	74.0	605034	51484	250	6.4
**Kenya**	23.0	101	264.4	66.1	57883	4769	630	24.8
**Kiribati**	29.6	106	1396.0	59.4	-	24974	-	28.2
**Kuwait**	30.0	583	1412.7	81.0	1575162	94926	121	25.35
**Kyrgyzstan**	26.2	445	519.6	74.2	284910	27577	533	1.55
**Laos**	22.6	88	814.6	68.5	129279	13529	1834	22.8
**Latvia**	25.8	3010	1189.1	75.4	3287714	144675	667	5.6
**Lebanon**	27.8	1516	2037.5	76.4	707607	103494	661	16.4
**Lesotho**	24.9	321	448.0	50.7	173523	11817	788	11.85

**Liberia**	24.0	56	154.4	64.1	26658	1131	2391	25.3
**Libya**	28.4	910	327.2	75.8	315837	54633	56	21.8
**Lithuania**	26.6	3314	1292.0	76.0	2718294	189169	656	6.2
**Luxembourg**	26.5	1590	6330.9	82.4	6180878	151394	934	8.65
**Madagascar**	21.1	48	404.2	65.3	12862	1646	1513	22.65
**Malawi**	22.8	131	241.1	65.6	26513	3285	1181	21.9
**Malaysia**	25.3	1048	441.2	74.7	1349827	82536	2875	25.4
**Maldives**	25.1	533	560.9	79.6	3700009	169626	1972	27.65
**Mali**	22.8	34	316.9	62.8	28102	918	282	28.25
**Malta**	27.2	1414	1527.9	81.9	2732197	95858	560	19.2
**Mauritania**	24.8	202	30.3	68.4	141307	8310	92	27.65
**Mauritius**	25.6	759	542.5	74.1	281291	18025	2041	22.4
**Mexico**	28.1	2456	327.1	76.0	107342	30050	758	21.0
**Moldova**	26.7	2838	1747.2	73.3	663403	92639	450	9.45
**Mongolia**	26.0	646	1982.5	68.1	1198853	115433	241	-0.7
**Montenegro**	26.0	4295	1100.8	75.9	1772960	256497	-	10.55
**Morocco**	25.6	426	682.3	73.0	298848	25376	346	17.1
**Mozambique**	22.3	67	501.9	58.1	37484	4931	1032	23.8
**Myanmar**	22.6	353	226.5	69.1	115400	9617	2091	13.05
**Namibia**	24.3	1533	298.4	64.6	352116	53489	285	19.95
**Nepal**	22.2	398	511.6	70.9	174195	27628	1500	8.1
**Netherlands**	25.4	1270	1459.9	81.8	1227550	174600	778	9.25
**New Zealand**	27.9	40	685.1	82.0	1206227	2705	1732	10.55
**Nicaragua**	26.9	33	327.8	75.0	-	2580	2280	24.9
**Niger**	21.7	12	118.8	63.3	8501	283	151	27.15
**Nigeria**	23.4	15	162.5	62.6	18935	1054	1150	26.8
**North Korea**	21.8	-	993.3	72.6	-	-	1054	5.7
**North Macedonia**	25.8	4412	2784.9	74.8	856865	106282	619	9.8
**Norway**	26.0	426	552.8	82.6	1795150	64643	1414	1.5
**Oman**	26.9	797	271.1	73.9	4706868	57562	125	25.6
**Pakistan**	23.8	133	363.2	65.6	109895	5686	494	20.2
**Palau**	29.4	329	-	-	21828100	218550	-	27.6
**Panama**	27.1	1841	219.3	79.3	1172489	109561	2928	25.4
**Papua New Guinea**	25.3	69	1689.3	65.3	27039	3916	3142	25.25
**Paraguay**	25.8	2555	384.5	75.8	322281	64000	1130	23.55
**Peru**	26.3	6280	97.7	79.9	750321	67356	1738	19.6
**Philippines**	23.2	525	1132.2	70.4	243702	25400	2348	25.85
**Poland**	26.4	3037	1363.1	78.3	805369	105390	600	7.85
**Portugal**	26.2	2110	1133.4	81.6	3291624	120934	854	15.15
**Qatar**	29.2	241	1020.3	77.2	1176549	87932	74	27.15
**Romania**	25.3	3411	1204.3	75.6	1001131	94463	637	8.8
**Russia**	26.5	2510	2295.0	73.2	1695504	70314	460	-5.1
**Rwanda**	22.0	108	94.0	69.1	334003	7591	1212	17.85
**Saint Kitts and Nevis**	29.7	780	-	-	1210957	52241	1427	24.5
**Saint Lucia**	29.6	1972	553.9	74.3	684875	71282	2301	25.5
**São Tomé and Príncipe**	24.8	323	-	70.4	128718	16591	3200	23.75
**Saudi Arabia**	28.5	253	1341.0	74.3	1057674	15480	59	24.65
**Senegal**	23.0	112	359.4	68.6	56095	4267	686	27.85

**Serbia**	25.8	1813	1898.6	75.9	933053	147770	-	10.55
**Seychelles**	26.8	1640	543.6	73.3	-	242312	2330	27.15
**Sierra Leone**	22.8	15	468.6	60.8	31576	792	2526	26.05
**Singapore**	23.7	207	851.2	83.2	3825919	46656	2497	26.45
**Slovakia**	26.5	3519	1500.9	78.2	1020852	148443	824	6.8
**Slovenia**	26.9	3111	2236.5	81.3	1190453	216751	824	8.9
**Somalia**	21.9	81	198.5	56.5	24136	1401	282	27.05
**South Africa**	27.3	1649	509.6	65.3	367863	54906	495	17.75
**South Korea**	23.9	278	1667.4	83.3	307838	-	1274	11.5
**South Sudan**	25.2	12	383.7	62.8	28102	1196	900	-
**Spain**	26.7	2181	1499.0	83.2	1415329	118321	636	13.3
**Sri Lanka**	23.0	763	254.6	76.9	281331	26930	1712	26.95
**Sudan**	25.2	107	339.9	69.1	12382	1012	250	26.9
**Suriname**	27.4	2223	511.7	71.5	360514	86509	2331	25.7
**Swaziland**	26.5	1180	91.7	57.7	387237	-	788	21.4
**Sweden**	25.8	1779	716.2	82.4	1619172	123981	624	2.1
**Switzerland**	25.3	1557	1489.8	83.9	1906925	137014	1537	5.5
**Syria**	28.1	172	2291.7	72.7	8050	2748	252	17.75
**Taiwan**	-	36	-	-	438321	705	-	-
**Tajikistan**	25.4	13	361.1	69.5	-	1735	691	2.0
**Tanzania**	23.1	12	181.8	67.3	-	426	1071	22.35
**Thailand**	24.1	352	837.4	77.7	246456	31353	1622	26.3
**Togo**	23.2	32	147.3	64.3	77241	3123	1168	27.15
**Tonga**	31.9	19	986.9	72.6	1136750	42103	-	25.25
**Trinidad and Tobago**	28.7	2648	682.5	76.1	395921	61070	2200	25.75
**Tunisia**	26.8	2341	1580.0	77.0	333503	60092	207	19.2
**Turkey**	27.8	1136	1770.7	78.6	1525812	107271	593	9.9
**Turkmenistan**	26.4	-	962.1	69.7	-	-	161	15.1
**Uganda**	22.0	75	195.7	66.7	48859	2710	1180	22.8
**Ukraine**	26.0	2490	1849.4	73.0	416578	83434	565	8.3
**United Arab Emirates**	29.0	228	748.5	76.1	12371211	74003	78	27.0
**United Kingdom**	27.3	2398	827.7	81.4	6567768	167421	1220	8.45
**United States**	28.5	2998	1016.6	78.4	2689323	155937	715	8.55
**Uruguay**	26.8	2045	899.4	77.1	1489570	115948	1300	17.55
**Uzbekistan**	26.1	48	495.9	73.0	40249	5775	206	12.05
**Vanuatu**	26.2	3	1069.2	65.3	72222	19	2000	23.95
**Venezuela**	27.2	201	396.2	73.9	118657	15606	2044	25.35
**Vietnam**	21.6	426	1049.6	73.7	781673	15937	1821	24.45
**Yemen**	25.8	69	423.8	66.6	8595	328	167	23.85
**Zambia**	22.6	206	145.4	62.5	165628	11445	1020	21.4
**Zimbabwe**	23.4	357	122.9	60.7	130395	12851	657	21.0
