# Supplementary material for: The impact of mean body mass index on reported mortality from COVID-19 across 181 countries
Source: Front Public Health. 2023 Mar 13;11:1106313. doi: 10.3389/fpubh.2023.1106313 (PMC10042264; doi:10.3389/fpubh.2023.1106313)
Supplement: Supplementary file 2 [file Data_Sheet_2.pdf]

Our World In Data + BMI

country	start_date	deaths	tests	life_expectancy	population	death_rate	test_rate	BMI
**Afghanistan**	2020-02-24	2189.0	-	64.83	41128772.0	0.062464	-	21.6
**Albania**	2020-02-25	1181.0	247526.0	78.57	2842318.0	0.489225	102.537	26.1
**Algeria**	2020-02-25	2756.0	-	76.88	44903228.0	0.072266	-	26.2
**Andorra**	2020-03-02	84.0	-	83.73	79843.0	1.263170	-	27.5
**Angola**	2020-03-20	405.0	-	61.15	35588996.0	0.014523	-	24.1
**Anguilla**	2020-03-28	0.0	-	81.88	15877.0	0.000000	-	-
**Antigua and Barbuda**	2020-03-13	5.0	-	77.02	93772.0	0.066424	-	28.4
**Argentina**	2020-01-01	43245.0	5126379.0	76.67	45510324.0	0.950224	112.642	27.7
**Armenia**	2020-02-03	2823.0	595876.0	75.09	2780472.0	1.116213	235.609	26.7
**Aruba**	2020-03-13	49.0	-	76.29	106459.0	0.573375	-	-
**Australia**	2020-01-26	909.0	11260636.0	83.44	26177410.0	0.037278	461.796	27.2
**Austria**	2020-02-25	7486.0	-	81.54	8939617.0	0.985966	-	25.4
**Azerbaijan**	2020-03-01	2641.0	-	73.0	10358078.0	0.305128	-	27.4
**Bahamas**	2020-03-16	170.0	-	73.92	409989.0	0.521881	-	28.8
**Bahrain**	2020-02-24	352.0	2355313.0	77.29	1472237.0	0.280606	1877.601	28.2
**Bangladesh**	2020-03-08	7559.0	3228434.0	72.59	171186368.0	0.054084	23.099	21.0
**Barbados**	2020-03-17	7.0	-	79.19	281646.0	0.031390	-	28.7
**Belarus**	2020-02-28	1424.0	-	74.79	9534956.0	0.177560	-	26.6
**Belgium**	2020-02-04	19528.0	6966831.0	81.63	11655923.0	1.847464	659.103	25.5
**Belize**	2020-03-23	248.0	-	74.62	405285.0	0.789219	-	28.9
**Benin**	2020-03-16	44.0	-	61.77	13352864.0	0.004147	-	23.4
**Bermuda**	2020-03-19	10.0	-	82.59	64207.0	0.198075	-	-
**Bhutan**	2020-03-06	0.0	281699.0	71.78	782457.0	0.000000	438.023	23.8
**Bolivia**	2020-03-11	9165.0	413924.0	71.51	12224114.0	0.927654	41.896	25.9
**Bonaire Sint Eustatius and Saba**	2020-04-02	3.0	-	77.79	27052.0	0.148270	-	-
**Bosnia and Herzegovina**	2020-03-05	4050.0	514380.0	77.4	3233530.0	1.518814	192.901	26.1
**Botswana**	2020-03-30	42.0	-	69.59	2630300.0	0.021117	-	24.7
**Brazil**	2020-02-26	195072.0	29244757.0	75.88	215313504.0	1.070183	160.439	25.9
**British Virgin Islands**	2020-03-28	1.0	-	79.07	31332.0	0.041904	-	-
**Brunei**	2020-03-09	3.0	-	75.86	449002.0	0.008211	-	26.2
**Bulgaria**	2020-03-08	7576.0	1149545.0	75.05	6781955.0	1.368238	207.610	26.0
**Burkina Faso**	2020-03-10	85.0	-	61.58	22673764.0	0.004623	-	22.1
**Burundi**	2020-03-31	2.0	-	61.58	12889583.0	0.000206	-	20.9
**Cambodia**	2020-01-27	0.0	-	69.82	16767851.0	0.000000	-	21.9
**Cameroon**	2020-03-06	448.0	-	59.29	27914542.0	0.019526	-	24.4
**Canada**	2020-01-23	15736.0	17965095.0	82.43	38454328.0	0.435460	497.145	27.2
**Cape Verde**	2020-03-20	113.0	-	72.98	593162.0	0.243126	-	24.7
**Cayman Islands**	2020-03-13	2.0	-	83.92	68722.0	0.036254	-	-
**Central African Republic**	2020-03-15	63.0	-	53.28	5579148.0	0.014164	-	22.4
**Chad**	2020-03-19	104.0	-	54.24	17723312.0	0.007463	-	22.3
**Chile**	2020-02-23	16608.0	6443459.0	80.18	19603736.0	0.991098	384.520	27.8
**China**	2020-01-22	4733.0	-	76.91	1425887360.0	0.003522	-	23.9
**Colombia**	2020-03-06	43213.0	8104617.0	77.29	51874028.0	1.013529	190.088	25.9
**Comoros**	2020-04-30	10.0	-	64.32	836783.0	0.017804	-	24.1
**Congo**	2020-03-15	108.0	-	64.57	5970430.0	0.022689	-	23.3
**Costa Rica**	2020-03-06	2185.0	427761.0	80.28	5180836.0	0.513125	100.455	26.9
**Cote d'Ivoire**	2020-03-11	137.0	258506.0	57.78	28160548.0	0.006019	11.358	23.6

**Croatia**	2020-02-25	3920.0	1019109.0	78.49	4030361.0	1.145179	297.720	25.5
**Cuba**	2020-03-12	146.0	1490767.0	78.8	11212198.0	0.016166	165.069	26.2
**Curacao**	2020-03-14	14.0	-	78.88	191173.0	0.091540	-	-
**Cyprus**	2020-03-08	120.0	1037630.0	80.98	896007.0	0.164039	1418.429	27.0
**Czechia**	2020-03-01	11580.0	4224539.0	79.38	10493990.0	1.320569	481.761	26.9
**Democratic Republic of Congo**	2020-03-11	591.0	-	60.68	99010216.0	0.007385	-	22.2
**Denmark**	2020-02-02	1298.0	10540209.0	80.9	5882259.0	0.241868	1964.055	25.3
**Djibouti**	2020-03-18	61.0	-	67.11	1120851.0	0.068974	-	23.3
**Dominica**	2020-03-22	0.0	-	75.0	72758.0	0.000000	-	27.0
**Dominican Republic**	2020-03-01	2414.0	876244.0	74.08	11228821.0	0.257274	93.386	26.7
**Ecuador**	2020-03-01	14034.0	704513.0	77.01	18001002.0	0.932992	46.837	27.0
**Egypt**	2020-02-14	7631.0	-	71.99	110990096.0	0.078178	-	29.2
**El Salvador**	2020-03-19	1327.0	621580.0	73.32	6336393.0	0.266342	124.757	27.4
**Equatorial Guinea**	2020-03-15	86.0	-	58.74	1674916.0	0.064403	-	25.6
**Eritrea**	2020-03-21	3.0	-	66.32	3684041.0	0.001043	-	20.5
**Estonia**	2020-01-06	229.0	640612.0	78.74	1326064.0	0.175090	489.802	25.5
**Eswatini**	2020-03-14	205.0	-	60.19	1201680.0	0.213243	-	26.5
**Ethiopia**	2020-03-13	1923.0	1800236.0	66.6	123379928.0	0.019416	18.176	20.6
**Faeroe Islands**	2020-03-04	0.0	-	80.67	53117.0	0.000000	-	-
**Falkland Islands**	2020-04-04	0.0	-	81.44	3801.0	0.000000	-	-
**Fiji**	2020-01-30	2.0	21365.0	67.44	929769.0	0.002337	24.962	27.2
**Finland**	2020-01-29	592.0	2505181.0	81.91	5540745.0	0.115722	489.704	25.9
**France**	2020-01-24	64644.0	35881338.0	82.66	67813000.0	1.017377	564.706	25.3
**French Polynesia**	2020-03-13	114.0	-	77.66	306292.0	0.463654	-	-
**Gabon**	2020-03-14	64.0	-	66.47	2388997.0	0.033487	-	25.5
**Gambia**	2020-03-17	124.0	-	62.05	2705995.0	0.057875	-	24.0
**Georgia**	2020-02-26	2505.0	371383.0	73.77	3744385.0	0.790245	117.159	27.2
**Germany**	2020-01-27	33071.0	-	81.33	83369840.0	0.427102	-	26.3
**Ghana**	2020-03-14	335.0	672364.0	64.07	33475870.0	0.012509	25.106	24.2
**Gibraltar**	2020-03-04	7.0	-	79.93	32677.0	0.258906	-	-
**Greece**	2020-02-26	4838.0	3382488.0	82.24	10384972.0	0.550294	384.738	27.3
**Greenland**	2020-03-16	0.0	-	71.7	56494.0	0.000000	-	-
**Grenada**	2020-03-22	0.0	-	72.4	125459.0	0.000000	-	27.0
**Guam**	2020-03-12	0.0	81134.0	80.07	171783.0	0.000000	586.365	-
**Guatemala**	2020-02-17	4813.0	609672.0	74.3	17843914.0	0.309593	39.217	26.5
**Guinea**	2020-03-13	81.0	-	61.6	13859349.0	0.007281	-	22.7
**Guinea-Bissau**	2020-03-25	45.0	-	58.32	2105580.0	0.027761	-	23.1
**Guyana**	2020-03-12	164.0	-	69.91	808727.0	0.251760	-	26.3
**Haiti**	2020-03-20	236.0	-	64.0	11585003.0	0.025998	-	24.1
**Honduras**	2020-03-11	3130.0	-	75.27	10432858.0	0.371203	-	26.4
**Hong Kong**	2020-01-23	148.0	5809821.0	84.86	7488863.0	0.021030	825.554	-
**Hungary**	2020-03-03	9537.0	2227027.0	76.88	9967304.0	1.152615	269.152	26.3
**Iceland**	2020-02-28	29.0	241454.0	82.99	372903.0	0.092461	769.827	25.9
**India**	2020-01-30	148994.0	172049274.0	69.66	1417173120.0	0.114209	131.881	21.9
**Indonesia**	2020-03-02	22138.0	-	71.72	275501344.0	0.096479	-	22.9
**Iran**	2020-02-19	55223.0	7620667.0	76.68	88550568.0	0.720335	99.405	26.2
**Iraq**	2020-02-24	12813.0	-	70.6	44496124.0	0.337957	-	28.0
**Ireland**	2020-02-29	2237.0	2375010.0	82.3	5023108.0	0.531208	563.981	27.5
**Isle of Man**	2020-03-20	25.0	-	81.4	84534.0	0.377429	-	-

**Israel**	2020-02-20	3325.0	7889431.0	82.97	9449000.0	0.407744	967.480	26.3
**Italy**	2020-01-31	74159.0	26598607.0	83.51	59037472.0	1.368624	490.884	26.0
**Jamaica**	2020-03-11	302.0	-	74.47	2827382.0	0.132158	-	27.4
**Japan**	2020-01-22	3492.0	4486717.0	84.63	123951696.0	0.029892	38.407	22.6
**Jordan**	2020-03-03	3834.0	3175555.0	74.53	11285875.0	0.409230	338.949	28.9
**Kazakhstan**	2020-03-13	2749.0	5539512.0	73.6	19397998.0	0.176540	355.746	27.4
**Kenya**	2020-03-06	1670.0	-	66.7	54027484.0	0.037607	-	23.0
**Kuwait**	2020-02-24	934.0	1254832.0	75.49	4268886.0	0.256782	344.988	30.0
**Kyrgyzstan**	2020-03-18	1355.0	-	71.45	6630621.0	0.258991	-	26.2
**Laos**	2020-03-24	0.0	-	67.92	7529477.0	0.000000	-	22.6
**Latvia**	2020-01-06	635.0	877015.0	75.29	1850654.0	0.347888	480.477	25.8
**Lebanon**	2020-02-21	1468.0	-	78.93	5489744.0	0.310840	-	27.8
**Lesotho**	2020-05-13	51.0	-	54.33	2305826.0	0.034798	-	24.9
**Liberia**	2020-03-17	83.0	-	64.1	5302690.0	0.019769	-	24.0
**Libya**	2020-03-04	1478.0	-	72.91	6812344.0	0.262219	-	28.4
**Liechtenstein**	2020-03-04	44.0	20939.0	82.49	39355.0	1.351259	643.046	-
**Lithuania**	2020-03-19	1800.0	1641368.0	75.93	2750058.0	0.832418	759.058	26.6
**Luxembourg**	2020-02-12	495.0	1651658.0	82.25	647601.0	0.863750	2882.060	26.5
**Macao**	2020-01-22	0.0	-	84.24	695180.0	0.000000	-	-
**Madagascar**	2020-03-20	261.0	-	67.04	29611718.0	0.011249	-	21.1
**Malawi**	2020-03-29	189.0	-	64.26	20405318.0	0.012205	-	22.8
**Malaysia**	2020-01-24	471.0	4317441.0	76.16	33938216.0	0.014811	135.770	25.3
**Maldives**	2020-03-08	48.0	306372.0	78.92	523798.0	0.112242	716.410	25.1
**Mali**	2020-03-25	269.0	-	59.31	22593598.0	0.015465	-	22.8
**Malta**	2020-03-05	219.0	514757.0	82.53	533293.0	0.497972	1170.477	27.2
**Marshall Islands**	2020-05-07	0.0	-	73.7	41593.0	0.000000	-	29.2
**Mauritania**	2020-03-14	347.0	-	64.92	4736146.0	0.091583	-	24.8
**Mauritius**	2020-03-18	10.0	-	74.99	1299478.0	0.009753	-	25.6
**Mexico**	2020-01-01	125807.0	3460436.0	75.05	127504120.0	0.986690	27.140	28.1
**Moldova**	2020-03-08	2985.0	-	71.9	3272993.0	1.117058	-	26.7
**Monaco**	2020-02-29	3.0	-	86.75	36491.0	0.098063	-	-
**Mongolia**	2020-03-10	1.0	600984.0	69.87	3398373.0	0.000363	218.069	26.0
**Montenegro**	2020-03-17	682.0	-	76.88	627082.0	1.373583	-	26.0
**Montserrat**	2020-03-18	1.0	-	74.16	4413.0	0.287188	-	-
**Morocco**	2020-02-07	7388.0	4457349.0	76.68	37457976.0	0.219483	132.419	25.6
**Mozambique**	2020-03-22	166.0	271947.0	60.85	32969520.0	0.006471	10.601	22.3
**Myanmar**	2020-03-27	2682.0	1818260.0	67.13	54179312.0	0.064761	43.905	22.6
**Namibia**	2020-03-14	205.0	209155.0	63.71	2567024.0	0.099824	101.847	24.3
**Nepal**	2020-01-25	1856.0	1932477.0	70.78	30547586.0	0.065034	67.714	22.2
**Netherlands**	2020-02-27	11459.0	6230581.0	82.28	17564020.0	0.773152	420.385	25.4
**New Caledonia**	2020-03-19	0.0	-	77.55	289959.0	0.000000	-	-
**New Zealand**	2020-02-28	26.0	1405854.0	82.29	5185289.0	0.005961	322.346	27.9
**Nicaragua**	2020-03-19	165.0	-	74.48	6948395.0	0.030200	-	26.9
**Niger**	2020-03-20	104.0	-	62.42	26207982.0	0.005064	-	21.7
**Nigeria**	2020-02-28	1289.0	948048.0	54.69	218541216.0	0.007013	5.158	23.4
**North Korea**	2020-04-02	0.0	26244.0	72.27	26069416.0	0.000000	1.346	21.8
**North Macedonia**	2020-02-26	2503.0	402103.0	75.8	2093606.0	1.412213	226.870	25.8
**Northern Mariana Islands**	2020-04-04	0.0	27096.0	76.74	49574.0	0.000000	736.164	-
**Norway**	2020-02-12	436.0	2737139.0	82.4	5434324.0	0.090663	569.170	26.0

**Oman**	2020-02-24	1499.0	-	77.86	4576300.0	0.384432	-	26.9
**Pakistan**	2020-02-25	10176.0	6696068.0	67.27	235824864.0	0.050806	33.432	23.8
**Palestine**	2020-03-05	1400.0	-	74.05	5250076.0	0.323362	-	-
**Panama**	2020-03-09	4022.0	1302483.0	78.51	4408582.0	1.121191	363.086	27.1
**Papua New Guinea**	2020-03-20	9.0	-	64.5	10142625.0	0.001132	-	25.3
**Paraguay**	2020-03-07	2262.0	560389.0	74.25	6780745.0	0.407227	100.887	25.8
**Peru**	2020-03-06	93070.0	5522513.0	76.74	34049588.0	3.325596	197.332	26.3
**Philippines**	2020-01-30	9244.0	6408076.0	71.23	115559008.0	0.086898	60.239	23.2
**Poland**	2020-03-04	28554.0	7604343.0	78.73	39857144.0	0.865858	230.591	26.4
**Portugal**	2020-03-01	6906.0	5681817.0	82.05	10270857.0	0.804661	662.024	26.2
**Puerto Rico**	2020-03-01	0.0	363762.0	80.1	3252412.0	0.000000	133.846	-
**Qatar**	2020-02-29	245.0	-	80.23	2695131.0	0.108432	-	29.2
**Romania**	2020-02-26	15767.0	4479391.0	76.05	19659270.0	0.947362	269.145	25.3
**Russia**	2020-01-31	56271.0	91123132.0	72.58	144713312.0	0.423667	686.070	26.5
**Rwanda**	2020-03-14	92.0	725804.0	69.02	13776702.0	0.008347	65.854	22.0
**Saint Helena**	2020-09-07	0.0	-	80.56	5401.0	0.000000	-	-
**Saint Kitts and Nevis**	2020-03-25	0.0	-	76.23	47681.0	0.000000	-	29.7
**Saint Lucia**	2020-03-14	5.0	-	76.2	179872.0	0.034747	-	29.6
**Saint Pierre and Miquelon**	2020-04-05	0.0	-	81.07	5885.0	0.000000	-	-
**Saint Vincent and the Grenadines**	2020-03-14	0.0	-	72.53	103959.0	0.000000	-	27.3
**Samoa**	2020-11-18	0.0	-	73.32	222390.0	0.000000	-	31.7
**San Marino**	2020-02-29	59.0	-	84.97	33690.0	2.088923	-	-
**Sao Tome and Principe**	2020-04-06	17.0	-	70.39	227393.0	0.101441	-	24.8
**Saudi Arabia**	2020-03-02	6223.0	11046767.0	75.13	36408824.0	0.205217	364.290	28.5
**Senegal**	2020-02-28	410.0	277749.0	67.94	17316452.0	0.028150	19.070	23.0
**Serbia**	2020-02-26	3211.0	2296303.0	76.0	6871547.0	0.551976	394.738	25.8
**Seychelles**	2020-03-15	0.0	-	73.4	107135.0	0.000000	-	26.8
**Sierra Leone**	2020-03-31	76.0	-	54.7	8605723.0	0.011722	-	22.8
**Singapore**	2020-01-23	29.0	-	83.62	5637022.0	0.005475	-	23.7
**Slovakia**	2020-03-06	2138.0	3244664.0	77.54	5643455.0	0.460929	699.514	26.5
**Slovenia**	2020-02-02	2697.0	678496.0	81.32	2119843.0	1.394524	350.826	26.9
**Solomon Islands**	2020-10-12	0.0	-	73.0	724272.0	0.000000	-	25.5
**Somalia**	2020-03-16	130.0	-	57.4	17597508.0	0.009298	-	21.9
**South Africa**	2020-02-07	28469.0	6609208.0	64.13	59893884.0	0.528943	122.796	27.3
**South Korea**	2020-01-22	917.0	4809285.0	83.03	51815808.0	0.018778	98.481	23.9
**South Sudan**	2020-04-05	63.0	76232.0	57.85	10913172.0	0.007804	9.443	25.2
**Spain**	2020-02-01	50837.0	22859368.0	83.56	47558632.0	1.168146	525.268	26.7
**Sri Lanka**	2020-01-27	204.0	1250417.0	76.98	21832150.0	0.010061	61.667	23.0
**Sudan**	2020-03-13	1468.0	-	65.31	46874200.0	0.039014	-	25.2
**Suriname**	2020-03-14	122.0	10118.0	71.68	618046.0	0.246745	20.464	27.4
**Sweden**	2020-02-01	8727.0	4179271.0	82.8	10549349.0	0.904036	432.934	25.8
**Switzerland**	2020-02-25	7873.0	3288431.0	83.78	8740471.0	1.060563	442.981	25.3
**Syria**	2020-03-22	711.0	-	72.7	22125242.0	0.041301	-	28.1
**Taiwan**	2020-01-16	7.0	126987.0	80.46	23893396.0	0.000306	5.543	-
**Tajikistan**	2020-05-01	90.0	-	71.1	9952789.0	0.013527	-	25.4
**Tanzania**	2020-03-16	21.0	-	65.46	65497752.0	0.000404	-	23.1
**Thailand**	2020-01-04	61.0	1634168.0	77.15	71697024.0	0.000858	22.982	24.1
**Timor**	2020-03-22	0.0	-	69.5	1341298.0	0.000000	-	21.2
**Togo**	2020-03-04	68.0	179607.0	61.04	8848700.0	0.009288	24.532	23.2

**Trinidad and Tobago**	2020-03-14	127.0	-	73.51	1531043.0	0.103687	-	28.7
**Tunisia**	2020-03-04	4676.0	-	76.7	12356116.0	0.457381	-	26.8
**Turkey**	2020-03-11	20881.0	24464277.0	77.69	85341248.0	0.302735	354.686	27.8
**Turks and Caicos Islands**	2020-03-28	6.0	-	80.22	45726.0	0.172280	-	-
**Uganda**	2020-03-21	251.0	750583.0	63.37	47249588.0	0.006803	20.345	22.0
**Ukraine**	2020-03-03	19281.0	-	72.06	39701744.0	0.585019	-	26.0
**United Arab Emirates**	2020-01-29	669.0	20890341.0	77.97	9441138.0	0.076748	2396.537	29.0
**United Kingdom**	2020-01-30	94998.0	49127610.0	81.32	67508936.0	1.528646	790.529	27.3
**United States**	2020-01-22	350555.0	257894445.0	78.86	338289856.0	1.099516	808.886	28.8
**United States Virgin Islands**	2020-03-24	0.0	31459.0	80.58	99479.0	0.000000	409.315	-
**Uruguay**	2020-03-13	181.0	639198.0	77.91	3422796.0	0.065875	232.637	26.8
**Uzbekistan**	2020-03-15	614.0	-	71.72	34627648.0	0.022241	-	26.1
**Vanuatu**	2020-11-10	0.0	-	70.47	326744.0	0.000000	-	26.2
**Vatican**	2020-03-06	0.0	-	75.12	808.0	0.000000	-	-
**Venezuela**	2020-03-14	1028.0	-	72.06	28301700.0	0.045404	-	27.2
**Vietnam**	2020-01-23	35.0	-	75.4	98186856.0	0.000379	-	21.6
**Wallis and Futuna**	2020-10-19	0.0	-	79.94	11596.0	0.000000	-	-
**Yemen**	2020-04-10	610.0	-	66.12	33696612.0	0.024934	-	25.8
**Zambia**	2020-03-18	388.0	601003.0	63.89	20017670.0	0.024565	38.051	22.6
**Zimbabwe**	2020-03-20	363.0	216254.0	61.49	16320539.0	0.028386	16.911	23.4
